# Differences in Psychological Inflexibility Among Men With Erectile Dysfunction Younger and Older Than 40 Years: Web-Based Cross-Sectional Study

**DOI:** 10.2196/45998

**Published:** 2024-01-03

**Authors:** Junichi Saito, Hiroaki Kumano, Mohammad Ghazizadeh, Chigusa Shimokawa, Hideki Tanemura

**Affiliations:** 1 Comprehensive Research Organization Waseda University Saitama Japan; 2 Faculty of Human Sciences Waseda University Saitama Japan; 3 Logos Science Corp Tokyo Japan

**Keywords:** erectile dysfunction, acceptance and commitment therapy, psychological inflexibility, depression, anxiety, men, cross-sectional study, psychological, utility, psychosocial, men, therapy, impotence, erection

## Abstract

**Background:**

Psychological inflexibility is a core concept of acceptance and commitment therapy (ACT), which is a comprehensive, transdiagnostic interpretation of mental health symptoms. Erectile dysfunction (ED) is a condition that affects male sexual performance, involving the inability to achieve and maintain a penile erection sufficient for satisfactory sexual activity. Psychosocial factors primarily influence ED in men younger than 40 years, whereas biological factors are more likely to be the underlying cause in older men.

**Objective:**

This web-based cross-sectional study examined differences in depression, anxiety, and psychological inflexibility among men with ED younger and older than 40 years in a Japanese population.

**Methods:**

We used a web-based survey to gather data from various community samples. ED was assessed by the International Index of Erectile Function‐5 (IIEF-5) questionnaire, while depression, anxiety, and psychological inflexibility were evaluated by the Patient Health Questionnaire-9 (PHQ-9), General Anxiety Disorder-7 (GAD-7), Acceptance and Action Questionnaire-II (AAQ-II), Cognitive Fusion Questionnaire (CFQ), and Valuing Questionnaire–Obstacle Subscale (VQ-OB) questionnaires. The chi‐square test estimated the scores of PHQ-9 and GAD-7 among men with ED, comparing those younger than 40 years and those older than 40 years. Additionally, a two-way ANOVA was conducted with ED severity and age group as independent variables, assessing psychological inflexibility.

**Results:**

Valid responses from 643 individuals (mean age 36.19, SD 7.54 years) were obtained. Of these, 422 were younger than 40 years (mean age 31.76, SD 5.00 years), and 221 were older than 40 years (mean age 44.67, SD 2.88 years). There was a statistical difference in the prevalence of depression as judged by PHQ≥10 between men with ED younger and older than 40 years (*P*<.001). On the other hand, there was no difference in the prevalence of anxiety as judged by GAD≥10 (*P*=.12). The two-way ANOVA revealed that the interactions for CFQ (*P*=.04) and VQ-OB (*P*=.01) were significant. The simple main effect was that men with ED younger than 40 years had significantly higher CFQ (*P*=.01; *d*=0.62) and VQ-OB (*P*<.001; *d*=0.87) scores compared to those older than 40 years in moderate ED and severe ED. Additionally, it was found that men younger than 40 years with moderate to severe ED had significantly higher CFQ (*P*=.01; *d*=0.42) and VQ-OB (*P*=.02; *d*=0.38) scores compared to men younger than 40 years without ED. On the other hand, no interaction was found for AAQ-II (*P*=.16) scores.

**Conclusions:**

To the best of our knowledge, this web-based cross-sectional study is the first to examine the relationship between psychological inflexibility and ED. We conclude that men with moderate and severe ED younger than 40 years have higher psychological inflexibility and might be eligible for ACT.

## Introduction

The efficacy of acceptance and commitment therapy (ACT) has been evaluated in numerous randomized controlled studies exploring various targeted conditions [1]. There is supporting evidence for ACT across various physical illnesses (eg, chronic pain [2], diabetes [3], epilepsy [4], cancer [5], and irritable bowel syndrome [6]). Many of these studies use a transdiagnostic method to analyze psychological issues within individual health conditions. Psychological inflexibility is a core concept of ACT, which is a comprehensive, transdiagnostic interpretation of mental health symptoms [7]. Psychological inflexibility highlights two interrelated processes: cognitive fusion and experiential avoidance. Cognitive fusion represents the phenomenon by which individuals are influenced by the literal meaning of their thoughts instead of viewing them as transient internal states [8]. Experiential avoidance represents an attempt or desire to suppress unwanted internal experiences, such as emotions, thoughts, memories, and bodily sensations [9]. These processes are obstacles to one’s valued living activities, decreasing well-being [10].

Erectile dysfunction (ED) is a condition that affects male sexual performance, involving the inability to achieve and maintain a penile erection sufficient for satisfactory sexual activity [11]. Several reviews and clinical guidelines are available for ED. However, many of these approaches to assessing and treating ED purely from a medical perspective seldom address the psychosocial components of ED [12]. Pharmacological treatment alone does not respond to all the concurrent factors of ED, including anxiety, loss of self-confidence, depressed mood, difficulties in a couple’s communication, relationship disputes, or a partner’s sexual problems [13]. Recent systematic reviews have shown that combining phosphodiesterase-5 inhibitors with psychological treatment exhibits significant potential for treating ED [14].

There is a widespread assumption that psychosocial factors primarily influence ED in men younger than 40 years, whereas biological factors are more likely to be the underlying cause of ED in older men. Moore et al [15] showed different symptom patterns among patients with ED according to age groups. They reported that younger men had comparatively more significant depressive symptoms, with lower relationship satisfaction, more negative reactions from partners, and lower job satisfaction. Given these findings, it is possible that people younger than 40 years are more psychologically inflexible than those older than 40 years and that ACT is more effective for them. However, studies on ACT for ED remain limited, with only a few identified. Therefore, this cross-sectional study used assessments to evaluate depression, anxiety, and psychological inflexibility in men younger and older than 40 years. However, it is well known that ED prevalence varies across geographical groups [16,17]; therefore, it is essential to research ED etiology according to different racial, cultural, religious, and socioeconomic backgrounds. There might be many potential patients with ED in Japan, so we conducted a web-based survey for this study.

## Methods

### Participants

To gather data from a wide range of community samples, we used a web-based survey, conducted with the assistance of a marketing research service provider (Rakuten Insight, Inc) in Japan. Based on the International Index of Erectile Function‐5 (IIEF-5) cutoff point [18], participants of all severities were recruited to include a certain percentage of patients of all ages. All enrolled participants followed the following criteria: (1) male; (2) aged 20 to 50 years; and (3) married or living with a fixed sexual partner for more than 6 months. The exclusion criteria were as follows: (1) sexual dysfunction caused by Peyronie disease or other organic lesions of the external genitalia; (2) prostate cancer, hypertensive disease, cardiac disease, cerebrovascular disease, chronic kidney disease, and diabetes; and (3) a history of sertraline or other medicines that may influence erection and psychological symptoms.

Participants were first instructed that this survey would be administered anonymously, and their responses were not compulsory. Then, those participants who agreed to participate in this research responded to the surveys. Participants were given points to exchange for items within the survey company’s system as a reward.

### Ethical Considerations

This study was approved by the Waseda University Academic Research Ethical Review Committee (2019-363). The study protocol followed the guidelines for epidemiological studies in accordance with the Declaration of Helsinki.

### Measurements

#### International Index of Erectile Function-5 (IIEF-5)

The Japanese version of IIEF-5 is a 5-item self-report questionnaire designed to measure erectile function [18]. Items are rated on a 5-point Likert-type scale, ranging from 1 to 5. The total score can range from 5 to 25, with high scores meaning high erectile function. Based on the original validation studies, the total score can then be interpreted as suggesting “no ED” (22-25), “mild ED” (17-21), “mild-to-moderate ED” (12-16), “moderate ED” (8-11), and “severe ED” (5-7).

#### Patient Health Questionnaire-9 (PHQ-9)

The Japanese version of the Patient Health Questionnaire-9 (PHQ-9) is a 9-item self-report questionnaire designed to measure depression [19]. Items are rated on a 4-point Likert-type scale, ranging from 0 to 3. The total score can range from 0 to 27, with high scores meaning high depression. Based on the original validation studies, the total score can then be interpreted as suggesting no depression (0-4), mild depression (5-9), moderate depression (10-14), moderately severe depression (15-19), or severe depression (20-27). A cutoff score of 10 is suggested as indicating a possible diagnosis of depressive disorder.

#### Generalized Anxiety Disorder-7 (GAD-7)

The Japanese version of the Generalized Anxiety Disorder-7 (GAD-7) questionnaire is a 7-item self-report questionnaire designed to measure generalized anxiety disorder [20]. Items are rated on a 4-point Likert-type scale, ranging from 0 to 3. The total score can range from 0 to 21, with high scores meaning high anxiety. Based on the original validation studies, the total score can then be interpreted as suggesting no anxiety (0-4), mild anxiety (5-9), moderate anxiety (10-14), or severe anxiety (14-21). A cutoff score of 10 is suggested as indicating a possible diagnosis of generalized anxiety disorder.

#### Acceptance and Action Questionnaire-II (AAQ-II)

The Japanese version of the Acceptance and Action Questionnaire-II (AAQ-II) is a 7-item self-report questionnaire designed to measure experiential avoidance [21]. Items are rated on a 7-point Likert-type scale, ranging from 1 to 7. The total score can range from 7 to 49, with high scores meaning high experiential avoidance.

#### Cognitive Fusion Questionnaire (CFQ)

The Japanese version of the Cognitive Fusion Questionnaire (CFQ) is a 7-item self-report questionnaire designed to measure cognitive fusion [22]. Items are rated on a 7-point Likert-type scale, ranging from 1 to 7. The total score can range from 7 to 49, with high scores meaning high cognitive fusion.

#### Valuing Questionnaire–Obstacle Subscale (VQ-OB)

The Japanese version of the Valuing Questionnaire–Obstacle Subscale (VQ-OB) is a 5-item self-report questionnaire designed to measure obstruction of valued living [23]. Items are rated on a 7-point Likert-type scale, ranging from 0 to 6. The total score can range from 0 to 30, with high scores meaning high obstruction of valued living.

### Statistical Analysis

We used mean (SD) values to describe numerical data and counts and percentages to describe categorical data. The chi‐square tests estimated categorical data, and numerical data were estimated by *t* tests. A two-way ANOVA test was used to assess the differences in men with ED aged younger and older than 40 years regarding psychological inflexibility and the interaction between them. Post hoc tests were conducted using the Holm method to control for type I errors. Cohen *d* index was calculated as effect sizes, serving as standardized indicators unaffected by sample sizes. All tests were 2-tailed, and a statistical difference was assumed when the *P* value was <.05. All statistical analyses were conducted through IBM SPSS Statistics (version 25.0; IBM Corp).

## Results

We obtained valid responses from 643 individuals (mean age 36.19, SD 7.54 years). Of these, 422 were younger than 40 years (mean age 31.76, SD 5.00 years), and 221 were older than 40 years (mean age 44.67, SD 2.88 years). Table 1 shows the demographic characteristics of participants by age difference. No statistical difference was found in ED severity, phosphodiesterase-5 inhibitors use, and marriage status between men with ED younger and older than 40 years.

**Table 1:** Demographic characteristics of men with erectile dysfunction (ED) younger and older than 40 years.

Characteristics		<40 years of age (n=422)	≥40 years of age (n=221)	*P* value
Age (years), mean (SD)		31.76 (5.00)	44.67 (2.88)	<.001
**Marital status, n (%)**				.44
	Single	77 (18.25)	35 (15.84)	
	Married	345 (81.75)	186 (84.16)	
Duration of marriage (years), mean (SD)		4.87 (3.90)	10.43 (7.53)	<.001
**IIEF-5^a^ severity, n (%)**				.84
	No ED	84 (19.91)	49 (22.17)	
	Mild to mild-to-moderate ED	226 (53.55)	112 (50.68)	
	Moderate to severe ED	112 (26.54)	60 (27.15)	
**PDE-5^b^ use, n (%)**				.63
	Not using	337 (79.86)	180 (81.45)	
	Using	85 (20.14)	41 (18.55)	

^a^IIEF-5: International Index of Erectile Function‐5.

^b^PDE-5: phosphodiesterase-5 inhibitor.

The prevalence of depression as judged by PHQ≥10 among men younger than 40 years was 39.81% (168/422), and it was 24.89% (55/221) among those older than 40 years. There was a statistical difference in the prevalence of depression between the two groups (*P*<.001). In addition, the prevalence of anxiety, as judged by GAD≥10, was 27.25% (115/422) among men younger than 40 years, and it was 21.72% (48/221) among those older than 40 years. There was no difference in the prevalence of anxiety between men with ED in the two age groups (*P*=.12). Table 2 illustrates these results.

**Table 2:** Prevalence of depression and anxiety among men with erectile dysfunction younger and older than 40 years.

Questionnaires and characteristics		<40 years of age (n=422)	≥40 years of age (n=221)	*P* value
**PHQ-9^a^**				<.001
	No depression or mild depression (PHQ-9<10), n (%)	254 (60.19)	166 (75.11)	
	Prevalence of depression (PHQ-9≥10), n (%)	168 (39.81)	55 (24.89)	
**GAD-7^b^**				.13
	No anxiety or mild anxiety (GAD-7<10), n (%)	307 (72.75)	173 (78.52)	
	Prevalence of anxiety (GAD-7≥10), n (%)	115 (27.25)	48 (21.72)	

^a^PHQ-9: Patient Health Questionnaire-9.

^b^GAD-7: Generalized Anxiety Disorder-7.

**Figure 1:**
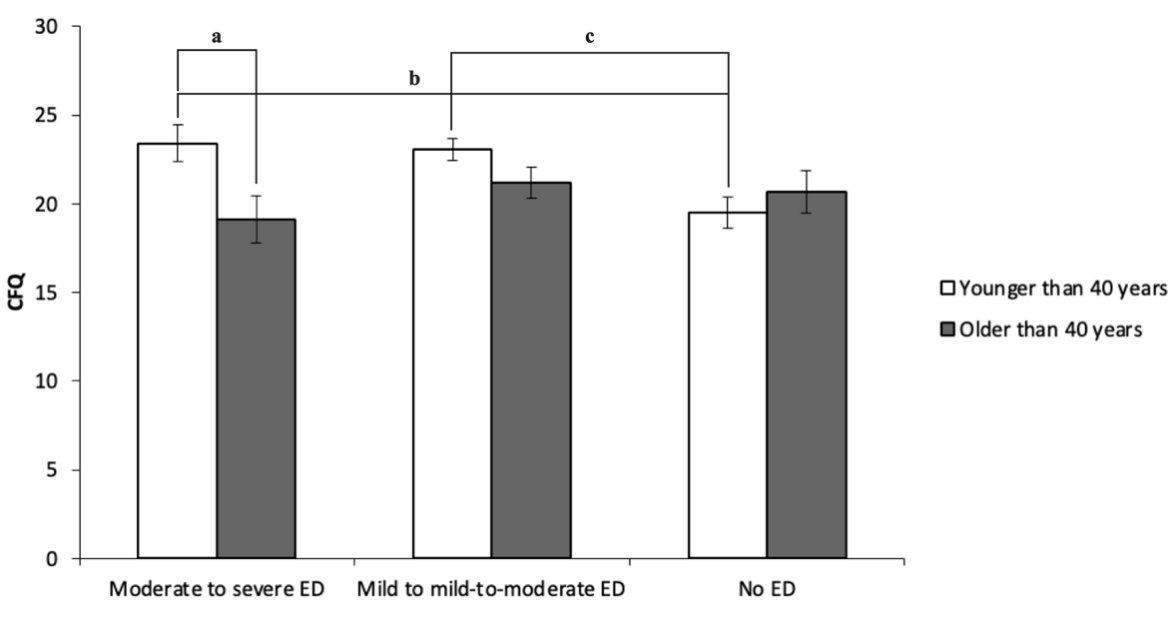
Results of the two-way ANOVA and the simple main effect of the Cognitive Fusion Questionnaire (CFQ). ED: erectile dysfunction. *P*=.01 for "a," "b," and "c".

**Figure 2:**
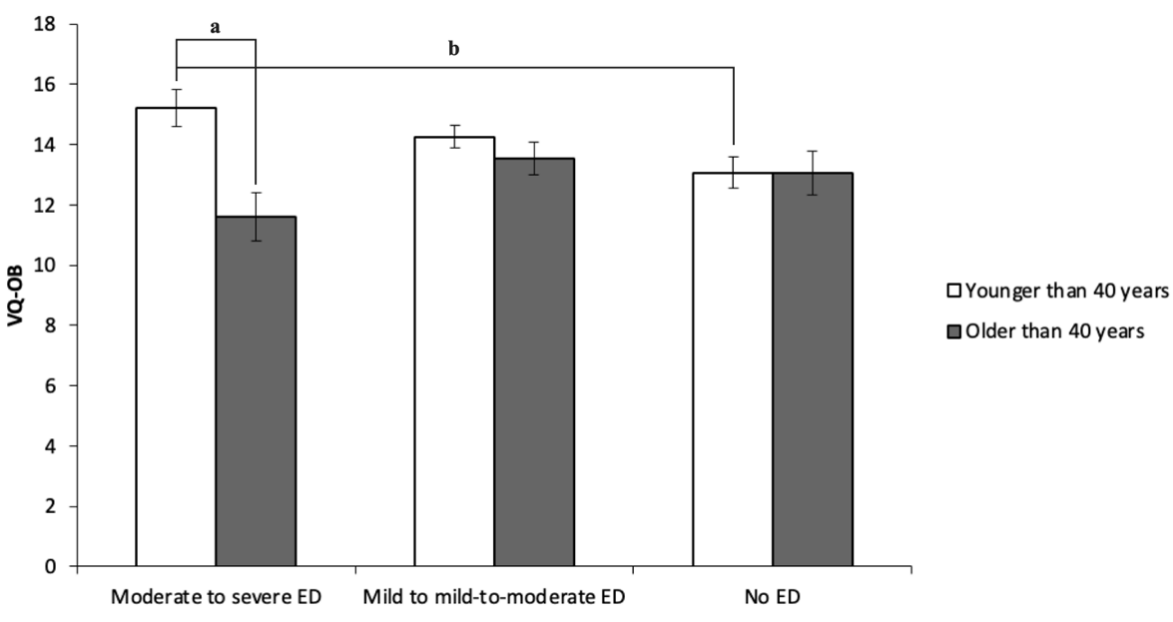
Results of the two-way ANOVA and the simple main effect of the Valuing Questionnaire–Obstacle Subscale (VQ-OB). ED: erectile dysfunction. *P*<.001 for "a" and *P*=.02 for "b".

The two-way ANOVA was performed with ED severity and age (<40 or >40 years) as independent variables and the scores of AAQ, CFQ, and VQ-OB as dependent variables. The results showed no significant differences in AAQ-II (*P*=.14), CFQ (*P*=.08), and VQ-OB (*P*=.30) scores attributed to ED severity. Moreover, no difference in ED severity or psychological inflexibility depending on the duration of the marriage was found. On the other hand, there were significant differences in the scores of CFQ (*P*=.04) and VQ-OB (*P*=.004) attributed to age. As the interactions were significant for CFQ (*P*=.04) and VQ-OB (*P*=.01) scores, the simple main effect was examined. It was found that men with ED younger than 40 years had significantly higher CFQ (*P*=.01; *d*=0.62) and VQ-OB (*P*<.001; *d*=0.87) scores compared to those older than 40 years, in cases of moderate and severe ED. Additionally, it was found that men with moderate to severe ED younger than 40 years had significantly higher CFQ (*P*=.01; *d*=0.42) and VQ-OB (*P*=.02; *d*=0.38) scores compared to men with no ED younger than 40 years. These results are illustrated in Table 3 and Figures 1 and 2.

**Table 3:** Two-way ANOVA results of the influence of erectile dysfunction (ED) severity, age, and interaction on psychological inflexibility.

Parameters and factors		Sum of squares	Mean squares	*F* test (df)	*P* value
**AAQ-II^a^**					
	ED severity	281.60	140.80	1.91 (2,637)	.14
	Age	204.74	204.74	2.78 (1,637)	.10
	ED severity × age	267.27	133.64	1.81 (2,637)	.16
**CFQ^b^**					
	ED severity	433.63	216.82	2.49 (2,637)	.08
	Age	348.12	348.12	3.99 (1,637)	.04
	ED severity × age	534.92	267.46	3.07 (2,637)	.04
**VQ-OB^c^**					
	ED severity	74.56	37.28	1.19 (2,637)	.30
	Age	263.36	263.36	8.38 (1,637)	.004
	ED severity × age	250.82	125.41	3.99 (2,637)	.01

^a^AAQ-II: Acceptance and Action Questionnaire-II.

^b^CFQ: Cognitive Fusion Questionnaire.

^c^VQ-OB: Valuing Questionnaire–Obstacle Subscale.

## Discussion

### Principal Findings

This cross-sectional study evaluated depression, anxiety, and psychological inflexibility in men younger and older than 40 years with ED. There was no statistical difference in demographic characteristics between the two groups. The average age of the participants was 31.76 (SD 5.00) years in men younger than 40 years; the population was assumed to have mainly mild-to-moderate psychogenic ED. On the other hand, the average age of the participants was 44.67 (SD 2.88) years in men older than 40 years; the population was assumed to have mostly mild-to-moderate organic ED.

Depression was found in both groups. The results of our study were consistent with a previous study [15], which found that younger men had comparatively greater depressive symptoms. In contrast, the prevalence of anxiety was not different between the two age groups. One possible reason is that the anxiety in men with ED is not general anxiety but specific anxiety about sexual situations. Masters and Johnson [24] highlighted the central role of sexual performance anxiety in couples presenting with sexual dysfunction [24]. In treating sexual dysfunctions, Kaplan [25] emphasizes the importance of addressing specific sources of sexual anxiety, such as fear of failure and not pleasing one’s partner [25]. Although a Japanese version does not exist now, it may be necessary to use a questionnaire like the Erectile Performance Anxiety Index [26].

Men with ED younger than 40 years had significantly higher CFQ and VQ-OB scores than those older than 40 years in cases of moderate and severe ED. Furthermore, men with moderate-to-severe ED younger than 40 years had significantly higher CFQ and VQ-OB scores compared to men without ED. These results partly support our hypothesis that men younger than 40 years are more psychologically inflexible than those older than 40 years. Cognitive fusion might be the critical component of ACT for ED. For example, the fusion with sexual performance anxiety, such as “I might fail again,” makes it impossible to pay attention to the sexual partner, which results in erectile failure. It is also consistent with Barlow’s theory [27]. Barlow [27] proposed a model for the interaction of anxiety and cognitive interference. This model examines how anxiety and cognitive interference interact, particularly in a sexual context, where a lack of control over one’s arousal diverts attention from erotic arousal to physical arousal and the negative consequences associated with failure to attain an erection.

On the other hand, there were no significant differences in the scores of the AAQ-II, which might be related to psychometric issues with AAQ-II. To date, the most used self-report measure of psychological inflexibility, especially experience avoidance, has been the AAQ-II. There was no significant difference in ED severity and psychological inflexibility depending on the duration of the marriage. However, various issues regarding the AAQ-II have emerged from the existing literature [28]. The authors found that the AAQ-II faced challenges in distinguishing distress (like negative affect and neuroticism) from experiential avoidance. For clinical application, researchers have expanded the range of measures for psychological inflexibility. They have developed specific versions of the AAQ-II tailored to different populations or disorders, with currently over 20 available versions (examples include those for the workplace, tinnitus, irritable bowel syndrome, exercise, and epilepsy). The disorder-specific AAQ-II variants indicate greater incremental validity in their targeted areas than the general AAQ-II [29]. Thus, developing a questionnaire on ED-related psychological inflexibility might be necessary.

There are some limitations to this study. First, this study used a cross-sectional approach, indicating merely “associations” rather than “causality” between psychological inflexibility and ED. Further controlled experimental and longitudinal studies are essential to delve deeper into the impact of psychological inflexibility on ED. Second, in this study, no responses were obtained from the partners of men with ED. Including the partners in the assessment and treatment of ED is recommended. It is desirable to obtain responses from partners in future studies. Finally, the specific racial or ethnic and socioeconomic profiles of the participants may restrict the broader applicability of the findings. The study was also conducted during the COVID-19 epidemic, which may have influenced the results.

### Conclusions

To the best of our knowledge, this web-based cross-sectional study was the first to examine the relationship between psychological inflexibility and ED. We conclude that men with moderate and severe ED younger than 40 years have higher psychological inflexibility and might be eligible for the ACT. In addition, developing a Japanese version of the questionnaire is necessary to measure ED-related anxiety and psychological inflexibility.

## Abbreviations

AAQ-II: Acceptance and Action Questionnaire‐II
ACT: Acceptance and Commitment Therapy
CFQ: Cognitive Fusion Questionnaire
ED: Erectile Dysfunction
GAD-7: Generalized Anxiety Disorder‐7
IIEF-5: International Index of Erectile Function‐5
PHQ-9: Patient Health Questionnaire‐9
VQ-OB: Valuing Questionnaire–Obstacle Subscale

## Data Availability

The data sets generated and analyzed during this study are not publicly available due to the Waseda University Academic Research Ethical Review Committee’s data-sharing policy but are available from the corresponding author upon reasonable request.
